# Production and Characterization of Red Fruit Spirits Made from Red Raspberries, Blueberries, and Strawberries

**DOI:** 10.3390/foods13081187

**Published:** 2024-04-13

**Authors:** Mário Bezerra, Fernanda Cosme, Fernando M. Nunes

**Affiliations:** 1 Chemistry Research Centre-Vila Real (CQ-VR), Food and Wine Chemistry Laboratory, University of Trás-os-Montes and Alto Douro, 5000-801 Vila Real, Portugal; mariojbezerra02@gmail.com (M.B.); fcosme@utad.pt (F.C.); 2 Biology and Environment Department, School of Life Sciences and Environment, University of Trás-os-Montes and Alto Douro, 5000-801 Vila Real, Portugal; 3 Chemistry Department, School of Life Sciences and Environment, University of Trás-os-Montes and Alto Douro, 5000-801 Vila Real, Portugal

**Keywords:** red berries, red fruit wines, red fruit spirits, chemical composition, volatile compounds

## Abstract

This study demonstrated the feasibility of fermenting and distilling low-commercial-value red fruits such as red raspberries, blueberries, and strawberries to produce high-value red fruit spirits. The fermentation process was efficient, with all red berry wines achieving a notable ethanol conversion yield (46.33 to 66.31%), without the need for nutrient supplementation or fruit juice solid separation, which showed no significant effect on the quality of the final product. Small-scale copper Charentais alembic distillation of the fermented red fruit juices resulted in fruit spirits equivalent to 1%, 7%, and 2% of the initial volume for red raspberries, blueberries, and strawberries, respectively. Except for the blueberry spirit, which had a lower volatile compound concentration (79.4 g/hL, absolute alcohol), all the produced red fruit spirits complied with legislation, including ethanol (37.9–40.2% *v*/*v*) and methanol (22.8–877.9 g/hL, absolute alcohol) concentrations and exhibited favorable aromatic profiles. The findings highlight that fermentation and distillation are straightforward, consistent, and reproducible methods, enabling the production of high-quality red fruit spirits from economically viable red fruit sources. This presents a significant opportunity in the spirits market, offering versatile applications as low-alcohol options, base spirits, or, with re-distillation, high-alcohol spirits.

## 1. Introduction

Red berries have gained widespread recognition not only for their delicious taste but also for their array of health benefits, making them a staple in the modern diet. Known for their antioxidant-rich profiles and various phytonutrients, red berries have emerged as an important component in promoting overall wellness [1,2,3,4,5,6]. However, during production, low-caliber fruits or overripe fruits represent an economic loss to producers. Additionally, their perishable nature poses a challenge for producers seeking to extend their shelf life to reduce losses. Among red berries, red raspberries, strawberries, and blueberries are some of the most produced berries, reaching 886.5 ktons, 9175.4 ktons, and 1113.6 ktons for red raspberries, strawberries, and blueberries, respectively, in 2021 [7].

Globally, a staggering one-third of food produced for human consumption is lost or wasted, amounting to an alarming 1.3 billion tonnes annually [8]. This inefficiency in food utilization not only depletes valuable resources but also triggers a cascade of environmental repercussions, including soil erosion, deforestation, and pollution of water and air. Moreover, the processes involved in food production, storage, transportation, and waste management contribute to substantial greenhouse gas emissions [9]. In Europe, potential emission reductions are evident through concerted efforts to minimize food waste along the entire food production and consumption chain [10,11]. Recognizing the escalating environmental, social, and economic implications of food waste, governments, businesses, non-governmental organizations (NGOs), academics, and the general public are increasingly addressing this issue. An expanding body of evidence has quantified the volume of discarded food and the associated emissions throughout the food supply chain (e.g., [12,13]). Within the food supply chain, private households emerge as the primary contributors to food waste, constituting the largest fraction [14]. Nevertheless, food loss occurs all over the food chain, including post-harvest where certain items are either left unharvested or discarded immediately after harvesting due to their failure to meet the quality standards mandated by processors or the specified criteria of target markets, including factors like shape, size, color, and weight. In the case of blueberries, strawberries, and red raspberries, these “low-caliber” fruits represent 5.5%, 7%, and 10%, respectively, of the total processed fruit [15]. The urgent need to address this challenge is underscored by its profound impact on both the environment and society at large.

Alcoholic fermentation of these fruit juices is considered a solution, allowing for the creation of a range of appealing beverages with an extended shelf life and high market value. Besides the production of fruit wines, which poses some conservation challenges, their distillation for the production of fruit spirits had already been proposed since spirits are more stabilized due to their higher alcohol content. The production of fruit spirits is a trend that is gaining traction around the world. These distilled beverages, derived from a variety of berries such as raspberries, blackberries, cornel berries, currants, and blueberries, are becoming increasingly popular [16]. The appeal of these fruit spirits is often attributed to their distinct and nuanced flavor profiles [17,18,19,20,21,22,23,24]. The volatile profile, a critical determinant of the sensory characteristics of fruit spirits, is influenced by several factors. Firstly, the type and quality of fruit used play a key role, as they contribute unique aroma and flavor compounds to the final product [25]. Additionally, the conditions under which the alcoholic fermentation takes place can have a significant impact on the volatile composition, with variables such as temperature, yeast strains, and fermentation duration all playing a role. Although research in this area is still scarce and usually sugar supplementation is used, future efforts should focus on both the production and characterization of non-supplemented red berry spirits. By leveraging the inherent qualities of red berries and the expertise gained from traditional winemaking practices, the potential for creating exceptional red berry spirits is great [19,26,27,28].

The primary goal of this study was to evaluate the potential of “low caliber” and overripe strawberries, red raspberries, and blueberries, without sugar supplementation, in fruit spirits production. Traditionally, these berries might be overlooked or deemed unsuitable due to perceived flaws in appearance or size. Through the analysis of key chemical constituents, such as major volatile compounds, this study sought to elucidate the suitability and reliability of a simple fermentation and distillation process using “low caliber” berries for spirit production. It aimed to demonstrate the inherent qualities of these berry spirits and to serve as a base characterization for future product development.

## 2. Materials and Methods

### 2.1. Yeast and Yeast Preparation

Active dry yeast *Saccharomyces cerevisiae* subsp. bayanus (SAI, Paredes, Portugal) was selected for use in this study. Yeast activation was performed as recommended by the manufacturer, with a dilution of 1:10 (*m*/*v*) and allowing it to activate for 20 min. Inoculation was also carried out according to the manufacturer’s instructions, at a concentration of 0.2 g/L, giving 1 × 10^6^ cells/mL, which typically guarantees that the yeast is the main colonizer.

### 2.2. Preparation of Red Berry Juices for Fermentation

Since the main purpose of this study was to utilize red raspberries (*Rubus idaeus* L. cv. ‘Kweli’), blueberries (*Vaccinium corymbosum* cv. ‘Duke’), and strawberries (Fragaria × ananassa cv. ‘Camarosa’) of low industrial value, all the red berries were considered “low caliber” due to their size and shape. They were frozen and kindly donated by AgroAguiar-Agroindústria SA, Vila Pouca de Aguiar. The three types of red berries were crushed and pressed until a homogenous must was achieved using a commercial blender (Philips, Amsterdam, Netherlands) to obtain unfiltered juice, which was then characterized by standard enological parameters, namely Brix°, pH, total acidity, and yeast assimilable nitrogen, in duplicate. Additionally, to evaluate the influence of solid material on the fermentation process, all the juices were manually filtered through a 0.25 µm sieve to obtain filtered juice.

### 2.3. Alcoholic Fermentation

Red berry wines were produced in duplicate using traditional winemaking techniques. Initially, 1.5 L of each juice (both filtered and unfiltered) was allowed to ferment in a 5 L stainless steel tank at a constant temperature of 25 °C with regular shaking without sugar supplementation. The fermentation process was monitored by measuring the Brix° approximately 4 times a day, and fermentation was stopped upon reaching 3 constant Brix° readings. Fermentation additives such as diammonium phosphate (SAI, Paredes, Portugal) and yeast nutrients (autolyzed yeast, diammonium phosphate, cellulose, and thiamine) (SAI, Paredes, Portugal) were added at the beginning of fermentation, according to the manufacturer’s instructions (30 and 40 g/hL, respectively), to evaluate their influence on optimizing the fermentation process. At the end of fermentation, sulfur dioxide (SO_2_) (SOLFOX^®^6, SAI, Paredes, Portugal) was added to all the red berry wines at a concentration of 60 mg/L to prevent microbial growth. Subsequently, all wines were filtered again and stored at 4 °C until further analysis (around 2 days). For the production of red berry spirits, the same fermentation method was used but scaled up to 10 L of juice to yield a significant volume of red fruit wine. 

### 2.4. Characterization of Juices and Red Berry Wines

For the characterization of all the juices and red berry wines, the pH, Brix°, and total acidity were determined according to validated standard methods (OIV, 2015), while yeast assimilable nitrogen (YAN) was obtained using the formaldehyde method, as previously described in [29]. Briefly, after neutralization to pH 8.1, 2.5 mL of formaldehyde (37% *v*/*v*) was added to the sample (1:2.5). After 5 min, titration to pH 8.1 was performed using NaOH 0.05 N and YAN was calculated using the following formula: YAN (mg/L) = mL NaOH 0.05 N × 70. Ethanol yield (%) was calculated as follows:

(1) $$\mathrm{EtOH}\;\mathrm{in}\;\mathrm{final}\;\mathrm{wine}\;\left(g\right)=\mathrm{EtOH}\;\mathrm{in}\;\mathrm{final}\;\mathrm{wine}\;\left(\%\;v/v\right)\times 7.89$$

(2) Theoretical Yield =Total Sugars Consumed (g/L)×0.511

$$\mathrm{Ethanol}\;\mathrm{Yield}\;\left(\%\right)=\frac{\left(1\right)\;\mathrm{EtOH}\;\mathrm{in}\;\mathrm{final}\;\mathrm{wine}\;(g)}{\left(2\right)\;\mathrm{Theoretical}\;\mathrm{Yield}}\times 100$$

#### 2.4.1. Reducing Sugar Determination

Reducing sugars were analyzed by high-performance anion-exchange chromatography with pulsed amperometric detection (HPAEC-PAD) on a Dionex ICS 3000 system (Dionex, Sunnyvale, CA, USA) equipped with CarboPac PA-100 Guard and CarboPac PA-100 (250 mm × 4 mm) columns, as described by Vilela et al. [30]. An injection volume of 100 μL was used, and elution was performed with a linear gradient from 0 to 81 min, starting with 70% eluent A (2 mmol/L barium hydroxide) and 30% eluent B (150 mmol/L sodium hydroxide containing 2 mmol/L barium hydroxide) and ending with 30% eluent A, 30% eluent B, and 40% eluent C (1 mol/L sodium acetate containing 2 mmol/L barium hydroxide). After each analysis, the column was cleaned with 500 mmol/L sodium hydroxide containing 2 mmol/L barium hydroxide for 10 min and conditioned for 15 min to the initial conditions. The flow rate was 1.0 mL/min, and the column temperature was maintained at 35 °C. The electrochemical detector consisted of a Au working electrode, a Ag/AgCl reference electrode, and a Ti counter electrode. Standard curves were performed using glucose and fructose standards (Sigma, Livonia, MI, USA). 

#### 2.4.2. Ethanol Content Determination

Ethanol content was determined using gas chromatography–flame ionization detection (GC-FID) with an ethanol standard curve and n-propanol as an internal standard. Briefly, 5 mL of the internal standard was added to 50 mL of each sample, and the final volume was adjusted to 100 mL. The GC-FID analysis was carried out using a GC-Ultra gas chromatograph (Thermo Scientific, Waltham, MA, USA) equipped with a flame ionization detector (250 °C) and a fused silica capillary column of polyethylene glycol (Supelcowax^TM^ 10, Merck, Darmstadt, Germany) with a length of 30 m, inner diameter of 0.32 mm, and film thickness of 0.25 μm. Hydrogen was used as the carrier gas (3.40 cm^3^·min^−1^). Samples (~1 μL) were injected into the injector (250 °C) in split mode (split ratio: 1:10). The oven temperature program started at 35 °C (for 10 min), then increased at a rate of 5 °C·min^−1^ to 90 °C, followed by an increase at a rate of 15 °C·min^−1^ to 250 °C, which was held for an additional 6 min. 

### 2.5. Red Fruit Sprit Production and Characterization of Major Volatile Compounds 

The distillation process was carried out in duplicate using a copper Charentais alembic with a volume of 10 L, into which, 10 L of red berry wine was placed. The alembic was heated using a heating plate. Once the temperature of the red berry wine was raised to 98 °C, the distillation process started, and the distillate began to condense in the condenser. The heating plate was adjusted for a drop-out flow of the distillate. To establish the distillation curves, successive fractions of 100 mL were collected over time. Each spirit fraction (100 mL) collected during the distillation process was analyzed for alcoholic strength directly by electronic densimetry (OIV, 2014), using an electronic densimeter (model 5000 DMA, Anton Paar, Tokyo, Japan). Additionally, the content of the major volatile compounds, namely methanol, acetaldehyde, ethyl acetate, 3-methyl-butanol, and isobutanol, of each fraction were analyzed by gas chromatography–flame ionization detection (GC-FID) using the method described previously. However, for the internal standard, 100 µL of the internal standard (4-methyl-2-pentanol, Sigma, USA) was added to the distilled sample, resulting in a total volume of 10 mL. For comparison purposes, 5 commercial wine spirits used in Port wine production were included as a group, and their major volatile compound profiles were characterized using the same method, with the ethanol content assumed to be 77% *v*/*v*, as regulated by Regulation No. 84/2010 [31].

### 2.6. Statistical Analysis 

The results are presented as mean ± standard deviation. Physicochemical data were analyzed using Student’s *t*-test for comparisons between two independent samples and analysis of variance (ANOVA) for comparisons involving more than two independent samples. In addition, the Dunnett post hoc test was applied to the physicochemical data to identify significant differences using GraphPad Prism 8.1 software (Boston, MA, USA). Differences were considered statistically significant when the *p*-values were less than 0.05. Principal component analysis (PCA) was used to compare the profiles of the major volatile compounds of the spirits using Statistica 10 software (StatSoft, Tulsa, OK, USA).

## 3. Results and Discussion

### 3.1. Characterization of Red Fruit Juices and Fermentation

The juices were characterized in terms of Brix°, titratable acidity, yeast assimilable nitrogen, and reducing sugars (see Supplementary Figure S1 for HPAEC-PAD chromatograms) to determine their suitability for the fermentation process (Table 1). Blueberries had the most favorable characteristics for producing “grape-like” wine, having the highest concentration of reducing sugars, mainly glucose (104.56 g/L), and a balanced acidity. On the other hand, red raspberries showed the highest acidity with a total reducing sugar content of 108.63 g/L, while strawberries had the lowest reducing sugar content (102.90 g/L) and acidity. These fermentable sugar levels are in line with those described in the literature. Viljakainen et al. [32] reported an average total sugar content of 68.08 g/L for strawberry juice and 105.22 g/L for raspberry juice, while Li et al. [33] reported 56.88 and 116.53 g/L of total sugars in strawberry and blueberry juices, respectively.

**Figure 1:**
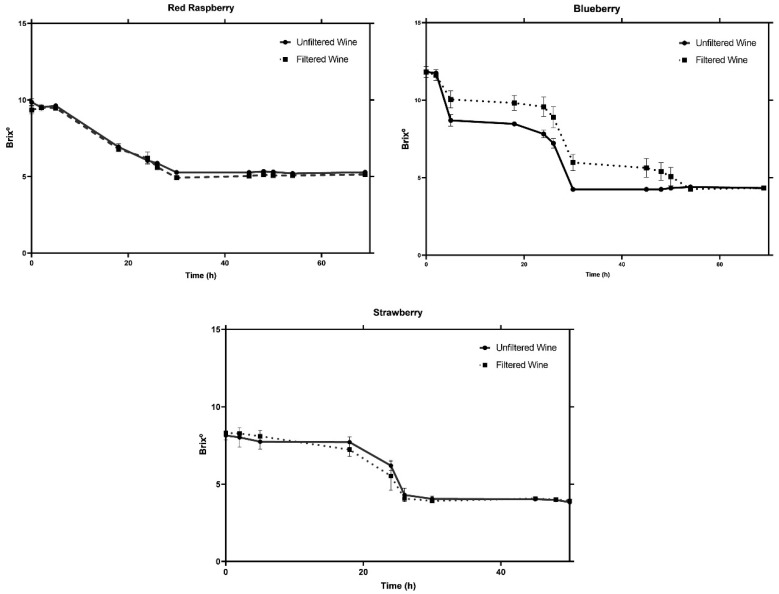
Fermentation curves of filtered (dashed line) and unfiltered (solid line) strawberry, blueberry, and red raspberry juices. Brix° was measured at each time point, and all fermentations were stopped after three constant readings.

Figure 1 illustrates the fermentation curves for each berry type, providing insights into the kinetics of the process. All fermentations were completed within a relatively short time frame of 70 h, underscoring the efficiency of the selected yeast strain and fermentation conditions. In particular, strawberries showed a particularly rapid fermentation, completing the process in approximately 50 h, in line with its initial lower fermentable sugar content.

Table 1 shows that the composition of the fruit wines mirrors that of the corresponding juices. Across the spectrum of the three berry juices, a consistent trend emerged: a decrease in pH coupled with an increase in acidity after fermentation. This phenomenon underscores the transformative impact of yeast metabolism during fermentation, where acids are generated as metabolic by-products [34]. 

As expected, the difference in ethanol content among the red fruit wines was directly related to the initial sugar concentration of each berry juice. Blueberries, characterized by their higher sugar content, naturally produced red fruit wines with the highest ethanol content upon fermentation. Conversely, although the strawberry and red raspberry juices started with similar total sugar levels, the slightly lower ethanol content of the red raspberry wines can be attributed to subtle differences in glucose concentrations. 

Tendentially, fruit wine production is often associated with sugar supplementation, resulting in final alcohol levels of 5–13% [35]. However, there is a notable market trend towards the consumption of low-alcohol wines, with volume consumption increasing by +8% in 2023 [36]. Consequently, the inherent low-alcohol characteristic of fruit wines produced from red fruits (without supplementation) can serve as a distinct advantage for this type of wine, with associated health attributes including a reduced calorie count, lower carbohydrate content, and often, the absence of residual sugar. Moreover, without sugar supplementation, there is less influence of fermentation aromas, and matrix-based profiles are more prominent in both fruit wines and fruit spirits (see Section 3.2).

Although the fermentation of the three red fruits apparently occurred without any observable problems, as the fermentable sugars were almost totally consumed (Table 1), ammonium diphosphate and fermentation nutrients were added to confirm that the juices had all the needed nutrients for optimal yeast activity. Nevertheless, no significant differences were observed during the fermentation process (results not shown), allowing us to conclude that the juices contained adequate levels of nutrients to perform the fermentation process. Another parameter evaluated was the fermentation in the presence of fruit solids. The analysis of the influence of the filtration step on the final product suggests that, although statistically significant differences were found, overall, this process does not significantly improve the overall quality of the red fruit wine regarding the analyzed parameters (Table 1). Filtration was found to have a positive but marginal influence on the ethanol content of the red fruit wines (0.5% *v*/*v*). When considering the first moment when the final Brix° value was reached, it is possible to see that filtration increased the speed of the fermentation step in strawberries. However, for the blueberry wine, the filtration step led to an increase of 39 h until the first constant Brix° value was reached. This result shows that the presence of solids may have some influence on yeast performance, but no clear trend was observed. For example, suspended solids may serve as a nutrient supply, mainly long-chain unsaturated fatty acids, and promote the adsorption of toxic fatty acids. Both factors combined serve as survival factors for yeast and can improve their efficiency. Therefore, by filtering the blueberry juice and then removing the suspended solids, the yeast efficiency may be reduced and the fermentation time may be longer [37].

### 3.2. Red Fruit Wine Distillation and Chemical Characterization of the Major Congeners in the Spirits

**Figure 2:**
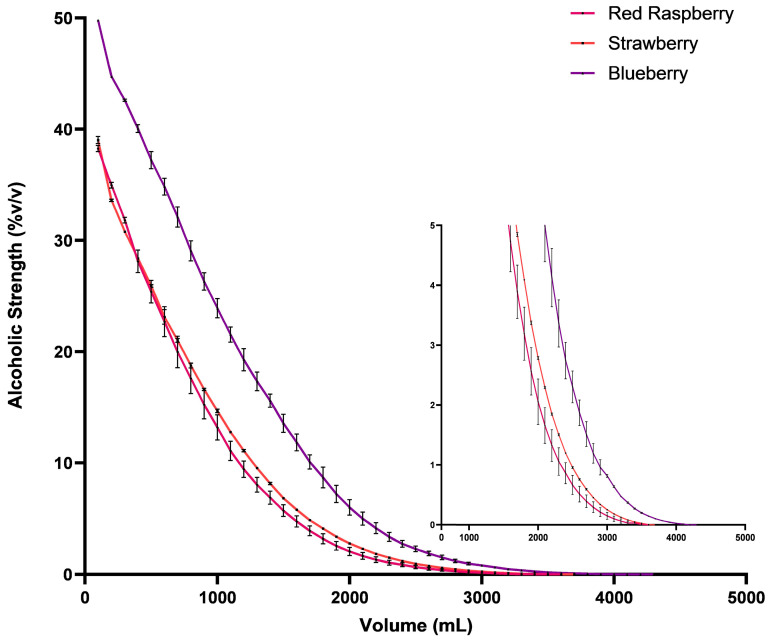
Distillation curves of red raspberry, blueberry, and strawberry wines are depicted. The curves represent the average (*n* = 2) ethanol content per 100 mL obtained by distillation for each fruit spirit. The small graph presents a zoomed-in curve, focusing on values below 5% *v*/*v* (see Supplementary Table S1 for individual mean ± SD values).

The red berry wines were distilled to evaluate their suitability for producing spirits and the quality of the obtained spirits. As expected from the initial ethanol content, the blueberry distillate had the highest initial ethanol content, approaching 50% *v*/*v*. It also yielded the largest total distillation volume until zero ethanol was reached in the distillate (3.9 L). The strawberry and red raspberry distillation exhibited remarkably similar behaviors, reflecting the characteristics of their respective wines, with initial ethanol contents around 39% and 38% *v*/*v*, respectively, and final volumes of approximately 3.5 L each (Figure 2).

**Figure 3:**
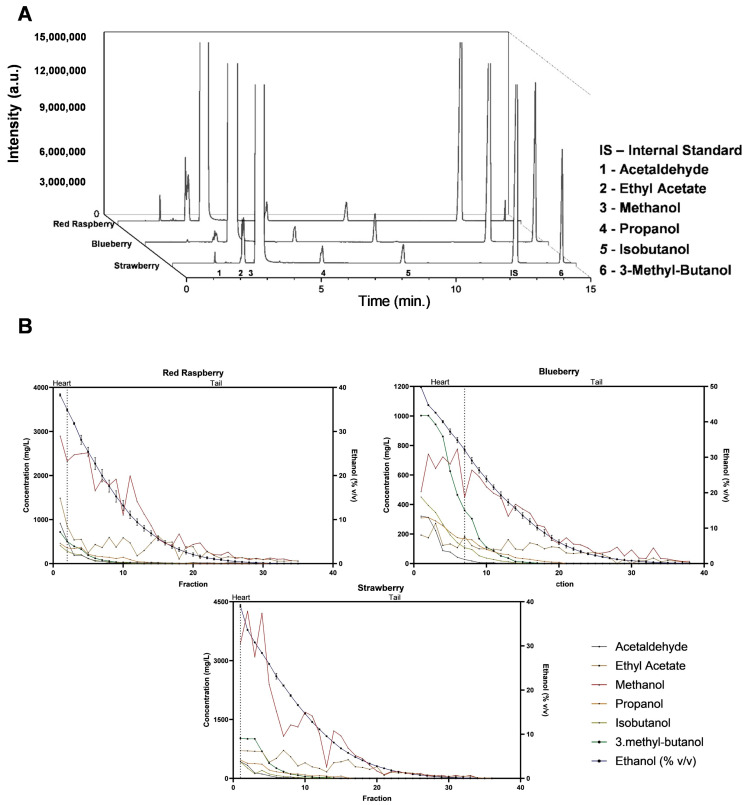
(**A**) GC-FID chromatograms of the major congeners in the heart fraction of red raspberry, blueberry, and strawberry spirits. Identified compounds are discriminated from 1 to 6, and internal standard (IS) corresponds to 4-methylpentanol, used as an IS. (**B**) Concentration (mg/L) of all identified compounds and respective ethanol content (% *v*/*v*) for each 100 mL distilled fraction (see Supplementary Table S2 for individual mean ± sd). The heart/tail cut is represented by the dashed line.

The main congeners present in each distillation fraction were evaluated by GC-FID, and methanol, acetaldehyde, propanol, isobutanol, and 3-methylbutanol were quantified. Distillation cuts for producing spirits are made based on several factors, such as ethanol concentration, sensory evaluation of the aromatic profile of the distillate, and theoretical distillation temperatures of different compounds. Therefore, these cuts are often subject to the expertise of the distiller [38,39]. Heads, characterized by high concentrations of volatile compounds such as methanol and acetaldehyde, are typically discarded to reduce the amount of toxic compounds and to avoid introducing unwanted flavors into the final product. Conversely, hearts, comprising the majority of the distillate, contain the desired aromatic compounds and flavors essential to the character of the spirit. Tails, with their distinct and often unpleasant odors, are also separated to maintain the integrity and quality of the final product [40,41]. In this case, the analysis of the distillation profile of each volatile compound (Figure 3) showed that all compounds, regardless of their type, were in the heart/tail stage [42]. Together with the theoretical equilibrium of ethanol–water mixtures and temperature [43], this suggests the absence of a head fraction and that the majority of the produced distillate is characteristic of tails. Therefore, the heart/tail cut was performed at volumes of 100, 700, and 200 mL for the red raspberry, blueberry, and strawberry distillates, respectively, which corresponded to 1%, 7%, and 2% of the initial 10 L volume.

According to EU Regulation 2019/787 [44], which regulates the production of fruit spirits, there are some quality parameters that need to be met. Initially, all distillations started below 86% *v*/*v* as required. Also, the alcoholic strength must be equal to or greater than 37.5% *v*/*v* for the fruit spirit designation, which was achieved. However, the content of volatile compounds, calculated as the sum of all the identified major volatile compounds, must exceed the minimum level of 200 g/hL aa (absolute alcohol). The red raspberry (771.47 g/hL aa) and strawberry (1659.06 g/hL aa) spirits exceeded this parameter well over the stipulated limit; however, the blueberry spirit (79.38 g/hL aa) failed to fulfill what is required by the regulation. In addition, the methanol levels in the spirits were found to be within the acceptable range set by the regulation, with the maximum acceptable level set at 1000 g/hL aa for blueberry and strawberry spirits, and 1200 g/hL aa for red raspberry spirts. The measured methanol concentrations were 343.85 g/hL aa, 22.80 g/hL aa, and 877.88 g/hL aa for the red raspberry, blueberry, and strawberry spirits, respectively. 

Regarding higher alcohols, for which the regulation does not specify minimum or maximum levels, the literature suggests that levels above 350 g/hL aa may indicate poor quality. In this study, the total alcohol content, calculated as the content of major volatile compounds minus acetaldehyde, ethyl acetate, and methanol, was found to be 179.31 g/hL aa, 45.02 g/hL aa, and 490.77 g/hL aa for the red raspberry, blueberry, and strawberry spirits, respectively. These values suggest that, although the strawberry spirit presented slightly higher levels, all three spirits had levels indicative of good quality and may be appealing to consumers.

**Figure 4:**
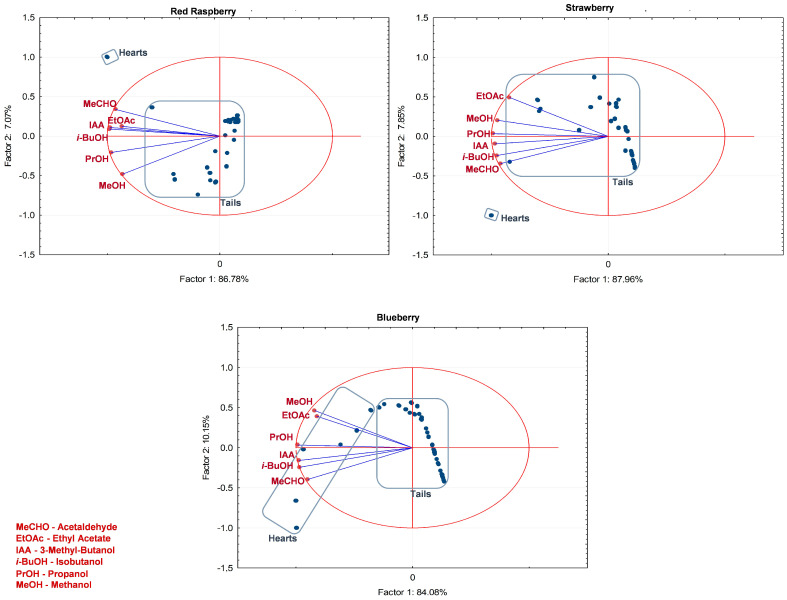
Principal component analysis for all fractions of the distillation process of red raspberry, blueberry, and strawberry spirits, along with their differentiation into heart and tail, and the influence of each identified major volatile compound of each fraction.

The cut performed for producing the red fruit spirits is supported by the results of Principal Component Analysis (PCA) of the concentration of congeners in the different distillation fractions (Figure 4). The first two principal components accounted for 93.9%, 95.8%, and 94.2% of the total variance in the original data set for the red raspberry, strawberry, and blueberry spirits, respectively. All variables correlated negatively with the first principal component, indicating that they all followed the same trend during the distillation process, decreasing as distillation proceeds. The hearts were clearly separated from the tails (Figure 4), showing a higher concentration of all congeners when compared to tails. This trend is in line with expectations, as the concentration of congeners gradually decreased during the distillation process in the order of heads, hearts, and tails [40].

The ethanol and congener concentrations of the obtained red fruit spirits, along with the average value of five commercial wine spirits for comparison, are shown in Table 2. Notable differences existed between the red fruit spirits, highlighting the distinct volatile profiles of the blueberry, strawberry, and red raspberry spirits. The blueberry spirit had the lowest congener concentration (79.38 g/hL aa), while the strawberry spirit had the highest (1659.06 g/hL aa). This difference underscores the influence of the berry type on the volatile composition of the resulting spirit. 

3-methyl-butanol, characterized for its alcoholic, sweet, and pungent aroma, is synthesized during fermentation through deamination and decarboxylation reactions from leucine [45,46]. Studies have reported a perception threshold of 30–65 mg/L for this compound [47,48]. All the red fruit spirits had concentrations of 3-methylbutanol well over the detection threshold (304.64, 107.34, and 1022.06 mg/L for the red raspberry, blueberry, and strawberry spirits, respectively). However, while the blueberry spirit was close to the threshold, the strawberry spirit stood out with a much higher concentration of this compound. This suggests that the strawberry spirit may have a fuller body than the raspberry and blueberry spirits, as low concentrations of amyl alcohols are typically associated with light-bodied spirits [49,50]. Similarly, González et al. [51] reached a similar conclusion regarding raspberry spirits, classifying them as light-bodied spirits by comparing the concentrations of 2-methyl-butanol and 3-methyl-butanol in raspberry and arbutus berry spirits. 

Propanol, known for its distinct and strong odor, is often associated with bacterial spoilage when present in high concentrations in distillates [49,52]. Research has shown that concentrations of propanol above the threshold of 800 mg/L [47] may indicate compromised quality due to improper storage conditions prior to distillation. The concentrations of propanol in the studied spirits were well below this perception threshold, with 203.9, 35.1, and 473.3 mg/L for the red raspberry, blueberry, and strawberry spirits, respectively. This suggests that the fermentations and their subsequent storage were conducted under appropriate conditions, reducing the risk of bacterial contamination and ensuring product quality [53].

Elevated levels of ethyl acetate in distilled beverages may indicate potential acetic spoilage [41,53,54] or may result from the improper separation of the head fraction during distillation [49]. This compound significantly influences the sensory characteristics of alcoholic beverages. In distillates, concentrations of ethyl acetate below 150 mg/L contribute to a pleasant aroma with fruity notes. However, concentrations above 150 mg/L can impart a vinegary taste and introduce spoilage nuances to the alcoholic beverage [52,53]. The blueberry spirit presented ethyl acetate levels below the established threshold with 24.2 mg/L, suggesting a potentially superior aromatic profile compared to the red raspberry and strawberry spirits, which had concentrations of 584.5 mg/L and 707.3 mg/L, respectively. However, it is important to recognize that aromatic characteristics are multifaceted and include a variety of compounds. Therefore, while higher levels of ethyl acetate in the red raspberry and strawberry distillates may be a cause for concern, they do not necessarily indicate a poor overall aromatic profile.

Acetaldehyde, a potent aroma compound found in several alcoholic beverages [55,56], is typically produced by the decarboxylation of pyruvate during alcoholic fermentation by yeast [57]. However, its production can also result from the metabolic activity of lactic or acetic acid bacteria [58]. At low levels, acetaldehyde contributes to a pleasant fruity aroma, but at higher concentrations, it emits a harsh and irritating odor [56]. Acetaldehyde is considered a potential source of carcinogenicity in alcoholic beverages [59,60]. As pointed out by Boffetta, Kaihovaara, Rudnai, Znaor, Lissowska, Swiatkowska, Mates, Pandics, and Salaspuro [60], fruit-based spirits tend to have high acetaldehyde levels. When comparing the results of this study to those obtained in [51] for raspberry spirits, it is evident that the acetaldehyde levels quantified in this study are significantly higher, except for the blueberry spirit.

González, Fernández, Castro, and Guerra [27] characterized blueberry spirits, and the heart fraction analyzed in this study presented very similar results regarding the commonly analyzed compounds when comparing the concentrations in g/hL aa. The ethanol content was similar to that in this study (45.26% and 39.03% *v*/*v*, respectively) and when comparing volatile compound concentrations, their study presented much higher values for all of the identified compounds. This discrepancy is evident when comparing their results with the ones obtained in our study regarding total alcohols (283.0 and 67.8 g/hL aa) and total volatiles (317.1 and 79.4 g/hL aa). Moreover, in their study, González, Fernández, Castro, and Guerra [27] also stated that acetaldehyde concentrations lower than 125 mg/L promote a pleasant aroma. This suggests that the blueberry spirit obtained in this study presented acetaldehyde concentrations associated with pleasant characteristics.

Methanol is a serious health hazard that can cause blindness and even death if inhaled or ingested [55]. This hazardous compound is produced by enzymatic processes catalyzed by pectin methylesterases that hydrolyze the esterified methoxyl groups of the pectin polymer found in crushed fruit [50,55,61]. Therefore, the presence of methanol in distilled spirits is closely related to the pectin content of the raw fruit material [62]. Therefore, the higher methanol content in fruit spirits is not surprising, as red berries have a higher pectin content compared to grapes. For example, Dreher [63] conducted a review in which the pectin content was estimated to be 35% of the total dietary fiber, as documented in the literature [64,65]. These results indicated a pectin content of 1.6 g/100 g, 0.8 g/100 g, and 0.7 g/100 g for raspberries, blueberries, and strawberries, respectively, in contrast to the 0.2 g/100 g reported for grapes. These values correlate with the methanol levels observed in the produced spirits, suggesting a higher methanol content in red berry spirits.

Cherry spirits have similarities to the red berry spirits examined in this study, where concentrations of key compounds such as propanol (61 g/hL aa), methanol (319.2 g/hL aa), and isobutanol (76.52 g/hL aa) were similar to those found in the red raspberry spirits, but higher than the blueberry and lower than the strawberry counterparts [66]. Conversely, 3-methylbutanol was detected at elevated levels (215.00 g/hL aa), comparable to the strawberry spirit, while the ethyl acetate (31.68 g/hL aa) and acetaldehyde (46.65 g/hL aa) levels were significantly lower than those observed in the red raspberry and strawberry spirits.

**Table 2:** Major congeners identified and quantified (g/hL aa) in the heart fraction of red raspberry, blueberry, strawberry, and commercial wine spirits, along with odor detection threshold (ODT) descriptors.

	Red Raspberry	Blueberry	Strawberry	Wine	ODT	Descriptor(s)
Ethanol (% *v*/*v*)	37.9 ± 0.1 ^c^	40.2 ± 0.1 ^a^	39.0 ± 0.1 ^b^	77.0 ± 0.0		
Methanol	343.9 ± 0.7 ^b^	22.8 ± 0.0 ^c^	877.9 ± 1.7 ^a^	48.0 ± 9.7	100 [67]	Sweet [67]
Acetaldehyde	94.5 ± 0.2 ^b^	5.5 ± 0.0 ^c^	109.5 ± 0.2 ^a^	1.2 ± 0.5	25 [53]	Nutty, sherry-like; green leaves [53]
Ethyl Acetate	153.9 ± 0.3 ^b^	6.0 ± 0.0 ^c^	180.9 ± 0.3 ^a^	16.3 ± 6.7	7.5 [48]	Fruity, sweet [48]
Propanol	53.7 ± 0.1 ^b^	8.7 ± 0.0 ^c^	121.1 ± 0.2 ^a^	13.3 ± 3.3	0.3 [68]	Alcohol, pungent [68]
Isobutanol	45.4 ± 0.1 ^b^	9.6 ± 0.0 ^c^	107.8 ± 0.2 ^a^	22.8 ± 5.8	0.2 [48]	Bitter, green, harsh [48]
3-Methyl-butanol	80.2 ± 0.1 ^b^	26.7 ± 1.1 ^c^	261.9 ± 0.0 ^a^	67.6 ± 15.2	30 [48]	Alcohol, floral [48]
Total Alcohols	179.3 ± 1.0 ^b^	45.0 ± 0.1 ^c^	490.8 ± 2.1 ^a^	103.7 ± 31.9		
Total Volatiles	771.5 ± 1.4 ^b^	79.4 ± 0.1^c^	1659.1 ± 2.6 ^a^	169.2 ± 36.0		

Results are presented as mean ± standard deviation (*n* = 2). For fruit spirits, values with different letters are significantly different (*p* < 0.05, ANOVA).

It is generally accepted that compounds with an Odor Activity Value (OAV), obtained by dividing their concentration by the Odor Detection Threshold (ODT), greater than 1 can directly influence the overall flavor. The OAV values of the detected congeners were calculated based on the quantitative results obtained by GC-FID, and the ODT values reported in the literature [48,53,67,68]. Of the six compounds analyzed in all matrices, all of them, except acetaldehyde in the wine spirits, presented OAV values greater than 1 (Table 2). These values also indicate that propanol and isobutanol are the main contributors to the aromatic profiles of the analyzed spirits. On the other hand, acetaldehyde was a minor contributor to the aromatic profile and had no influence on the blueberry and wine spirits. It is also noteworthy that when comparing the fruit matrices, the blueberry spirit showed a low influence from methanol, acetaldehyde, and ethyl acetate.

Recent investigations indicate that the positive impacts of moderate alcohol consumption on health are heavily reliant on the presence of bioactive constituents within alcoholic drinks [69]. These bioactive compounds play pivotal roles in biological functions such as scavenging free radicals, inhibiting lipid peroxidation, and reducing platelet aggregation [70]. A vast group of components including phenols, acids, pyrazines, sulfur compounds, terpenes, esters, furans, and peptides have been identified as beneficial to human health [71].

Further examinations indicate the pivotal role of esters like ethyl acetate in augmenting the response of GABA_A_ receptors (γ-aminobutyric acid type A receptors), possibly by integrating into the brain’s membrane lipids after crossing the blood–brain barrier [72,73]. For instance, the aroma of whisky has been demonstrated to enhance the electrical response of GABA_A_ receptors and prolong sleep duration in mice more effectively than an equivalent concentration of ethanol, indicating sedative effects attributable to minor constituents in whisky [74].

The balance between esters, particularly acetate esters, and higher alcohols significantly influences sensory attributes. While higher alcohols, as the most prevalent sensory compounds, enhance quality when present in appropriate concentrations and proportions, excessive levels (>400 mg/L) can adversely impact quality by imparting unpleasant flavors and causing health concerns such as headaches and intoxication [75,76]. Moreover, under optimal ratios of alcohols/acids/esters, higher alcohols minimally affect intoxication levels, while certain acids, esters, and other compounds may mitigate the associated symptoms. Various physiological and psychological manifestations like headaches, thirst, and cognitive function are linked to factors such as ethanol, aldehydes, acidity, and dosage [75] with headache predominantly arising from ethanol rather than other congeners and ingredients [77,78].

**Figure 5:**
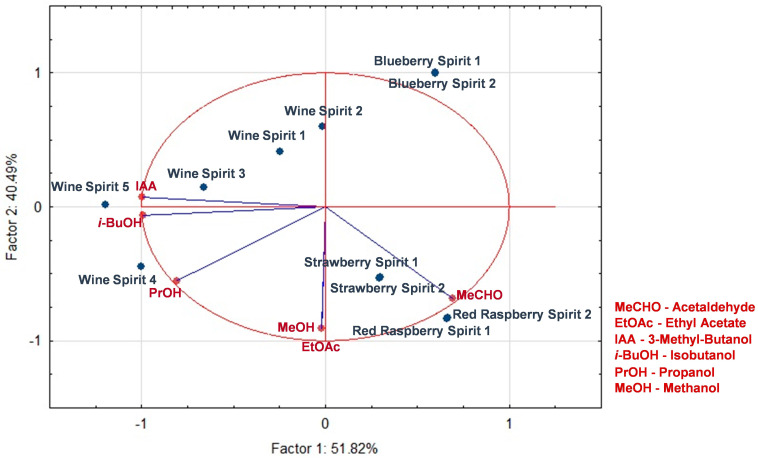
Principal component analysis showing the influence of the concentration of each identified major volatile compound, normalized for g/hL at 37.5% alcohol, on the differentiation of fruit spirits from wine spirits in the heart fraction of red raspberry, blueberry, and strawberry spirits.

For a comparison of the congener concentration of fruits spirits to that of the most common wine spirit, the concentrations of congeners in all spirits were corrected to 37.5% (*v*/*v*) ethanol. As can be observed in Figure 5, the PCA showed a higher variability in the wine spirits compared to each produced red fruit spirit. This is explained by the fact that the replicates for the red fruit spirits were made from the same raw material, while the different wine spirits were likely made from completely different raw materials and in different years. As expected, the results from the PCA reveal differences between the distilled fruit spirits and the distilled wine spirits. In particular, the fruit spirits had higher levels of acetaldehyde, and the raspberry and strawberry spirits also had higher levels of methanol and ethyl acetate. These differences are attributed mainly to the strict legislation that imposes maximum concentration levels of these compounds in wine spirts, lower than those allowed for fruit spirits, and to keep the product within those ranges, only the heart fraction is used [31]. On the other hand, the wine spirits presented higher levels of isobutanol, propanol, and 3-methylbutanol. High levels of 3-methylbutanol, a characteristic often found in wine spirits, were observed in the analyzed samples [79].

These results highlight the versatility of these fruits and their spirits, demonstrating their adaptability to the distillation process and their ability to produce spirits with distinctive characteristics. Of particular note is the relatively low ethanol content of these spirits, indicating their potential as a base for light drinks or liqueurs. This quality places them favorably in the growing market for low-alcohol beverages, meeting the evolving preferences of consumers seeking lighter options without compromising taste or quality [64].

## 4. Conclusions

The fermentation and distillation of “low-caliber” red raspberries, blueberries, and strawberries, which have low commercial value, allow for the production of fruit spirits with high market value. All red fruits were easily and effectively fermented without the need for juice supplementation with nutrients, resulting in a very good ethanol conversion yield (ranging from 46.33 to 66.31%). The distillation of the fermented red fruit juices allowed for the production of fruit spirits corresponding to 1%, 7%, and 2% of the initial volume for red raspberries, blueberries, and strawberries, respectively. With the exception of the blueberry spirit, due to its low concentration of volatile compounds (79.4 g/hL aa), all the spirits produced complied with legislation, especially regarding methanol concentrations (22.8–877.9 g/hL aa). Furthermore, the analysis of their major congeners indicated that all of them had a good and pleasant aromatic profile and different characteristics than wine spirts. The results showed that fermentation and distillation are simple, reliable, and reproductible methods, allowing for the production of high-quality spirits from low-cost fruit sources, which represents a significant opportunity in the spirits market. Red fruit spirits have the versatility to serve as low-alcohol alternatives, base spirits for other alcoholic beverages, or high-alcohol spirits if re-distillation is applied.

## Figures and Tables

**Table 1:** Standard enological characterization (Brix°, pH, titratable acidity, assimilable nitrogen, reducing sugars, and ethanol content and yield) of strawberry, blueberry, and red raspberry juices and wines, without the addition of fermentation additives.

Parameter	Juice	Fruit Wine Obtained from Unfiltered Juice	Fruit Wine Obtained from Filtered Juice
Red Raspberry			
pH	3.22 ± 0.01	2.82 ± 0.02 ^a^	2.86 ± 0.02 ^a^
Brix°	9.50 ± 0.22	5.27 ± 0.10 ^a^	5.07 ± 0.00 ^a^
Titratable acidity (g/L citric acid)	16.3 ± 0.1	18.1 ± 0.0 ^a^	18.2 ± 0.0 ^a^
Yeast assimilable nitrogen (mg/L)	184.33 ± 3.30	-	-
Total reducing sugars (g/L)	108.63 ± 0.04	0.39 ± 0.01 ^a^	0.38 ± 0.01 ^a^
Glucose (g/L)	47.88 ± 0.17	0.34 ± 0.01 ^a^	0.33 ± 0.01 ^a^
Fructose (g/L)	60.75 ± 0.21	0.05 ± 0.00 ^a^	0.05 ± 0.01 ^a^
Ethanol content (% *v*/*v*)	-	3.3 ± 0.1 ^b^	3.5 ± 0.2 ^a^
Ethanol yield (%)	-	46.33 ± 0.14 ^b^	49.52 ± 0.07 ^a^
Fermentation time (h)	-	30 ± 0.00 ^a^	30 ± 0.00 ^a^
Blueberry			
pH	3.43 ± 0.01	2.17 ± 0.01 ^a^	2.18 ± 0.01 ^a^
Brix°	12.30 ± 0.14	4.25 ± 0.02 ^a^	4.27 ± 0.00 ^a^
Titratable acidity (g/L citric acid)	12.1 ± 0.1	13.3 ± 0.1 ^b^	13.7 ± 0.2 ^a^
Yeast assimilable nitrogen (mg/L)	86.33 ± 3.30	-	-
Reducing sugars (g/L)	138.32 ± 0.05	0.16 ± 0.00 ^a^	0.14 ± 0.01 ^a^
Glucose (g/L)	104.56 ± 0.14	0.13 ± 0.00 ^a^	0.10 ± 0.02 ^a^
Fructose (g/L)	33.76 ± 0.09	0.03 ± 0.00 ^a^	0.04 ± 0.00 ^a^
Ethanol content (% *v*/*v*)	-	5.4 ± 0.0 ^b^	5.8 ± 0.1 ^a^
Ethanol yield (%)	-	59.86 ± 0.28 ^b^	64.84 ± 0.11 ^a^
Fermentation time (h)	-	30 ± 0.00 ^b^	69 ± 0.00 ^a^
Strawberry			
pH	3.15 ± 0.02	2.72 ± 0.01 ^a^	2.70 ± 0.03 ^a^
Brix°	8.67 ± 0.25	3.83 ± 0.03 ^a^	3.92 ± 0.05 ^a^
Titratable acidity (g/L citric acid)	10.3 ± 0.1	10.8 ± 0.0 ^a^	10.8 ± 0.0 ^a^
Yeast assimilable nitrogen (mg/L)	305.67 ± 6.60	-	-
Reducing sugars (g/L)	102.90 ± 0.06	0.41 ± 0.06 ^a^	0.39 ± 0.02 ^a^
Glucose	64.22 ± 0.33	0.32 ± 0.12 ^a^	0.27 ± 0.09 ^a^
Fructose	38.68 ± 0.27	0.09 ± 0.01 ^a^	0.12 ± 0.00 ^a^
Ethanol content (% *v*/*v*)	-	3.7 ± 0.1 ^b^	4.4 ± 0.1 ^a^
Ethanol yield (%)	-	54.98 ± 0.23 ^b^	66.31 ± 0.30 ^a^
Fermentation time (h)	-	30 ± 0.00 ^a^	26 ± 0.00 ^b^

All results are presented as mean ± standard deviation (*n* = 2). For fruit wines (unfiltered and filtered), values with different letters are significantly different (*t*-test—Student’s *t*-test).

## Data Availability

The original contributions presented in the study are included in the article/Supplementary Material, further inquiries can be directed to the corresponding author.

## Supplementary Materials

The following supporting information can be downloaded at: https://www.mdpi.com/article/10.3390/foods13081187/s1, Figure S1: HPAEC-PAD chromatograms of strawberry, blueberry, and red raspberry juices (dilution: 1:500) and wines (dilution: 1:2). Glucose (1) and fucose (2) were identified by comparison with commercial pure standards; Table S1: Ethanol concentrations (% *v*/*v*) of red raspberry, blueberry, and strawberry were determined for each 100 mL distilled fraction until 0.00% *v*/*v* was reached. The values are represented as mean ± standard deviation (*n* = 2) of the ethanol content; Table S2: Concentrations (mg/L) of major congeners and ethanol content (% *v*/*v*) of red raspberry, blueberry, and strawberry spirits in each 100 mL distilled fraction. The values are represented as mean ± standard deviation (*n* = 2), and the distillation cuts are represented as “heart” or “tail” fraction.
