# Supplementary material for: Production and Characterization of Red Fruit Spirits Made from Red Raspberries, Blueberries, and Strawberries
Source: Foods. 2024 Apr 13;13(8):1187. doi: 10.3390/foods13081187 (PMC11048981; doi:10.3390/foods13081187)
Supplement: Supplementary file 1 [file foods-13-01187-s001.zip › foods-2946081-supplementary.pdf]

Article

Production and Characterization of Red Fruit Spirits Made from Red Raspberries, Blueberries, and Strawberries

Mário Bezerra[1], Fernanda Cosme[1,2], Fernando M. Nunes[1,3*]

[1] Chemistry Research Centre-Vila Real (CQ-VR), Food and Wine Chemistry Laboratory, University of Trás-os-Montes and Alto Douro, 5000-801 Vila Real, Portugal

[2] Biology and Environment Department, School of Life Sciences and Environment, University of Trás-os-Montes and Alto Douro, 5000-801 Vila Real, Portugal

[3] Chemistry Department, School of Life Sciences and Environment, University of Trás-os-Montes and Alto Douro, 5000-801 Vila Real, Portugal

*Correspondence: fnunes@utad.pt;

Supplementary Material

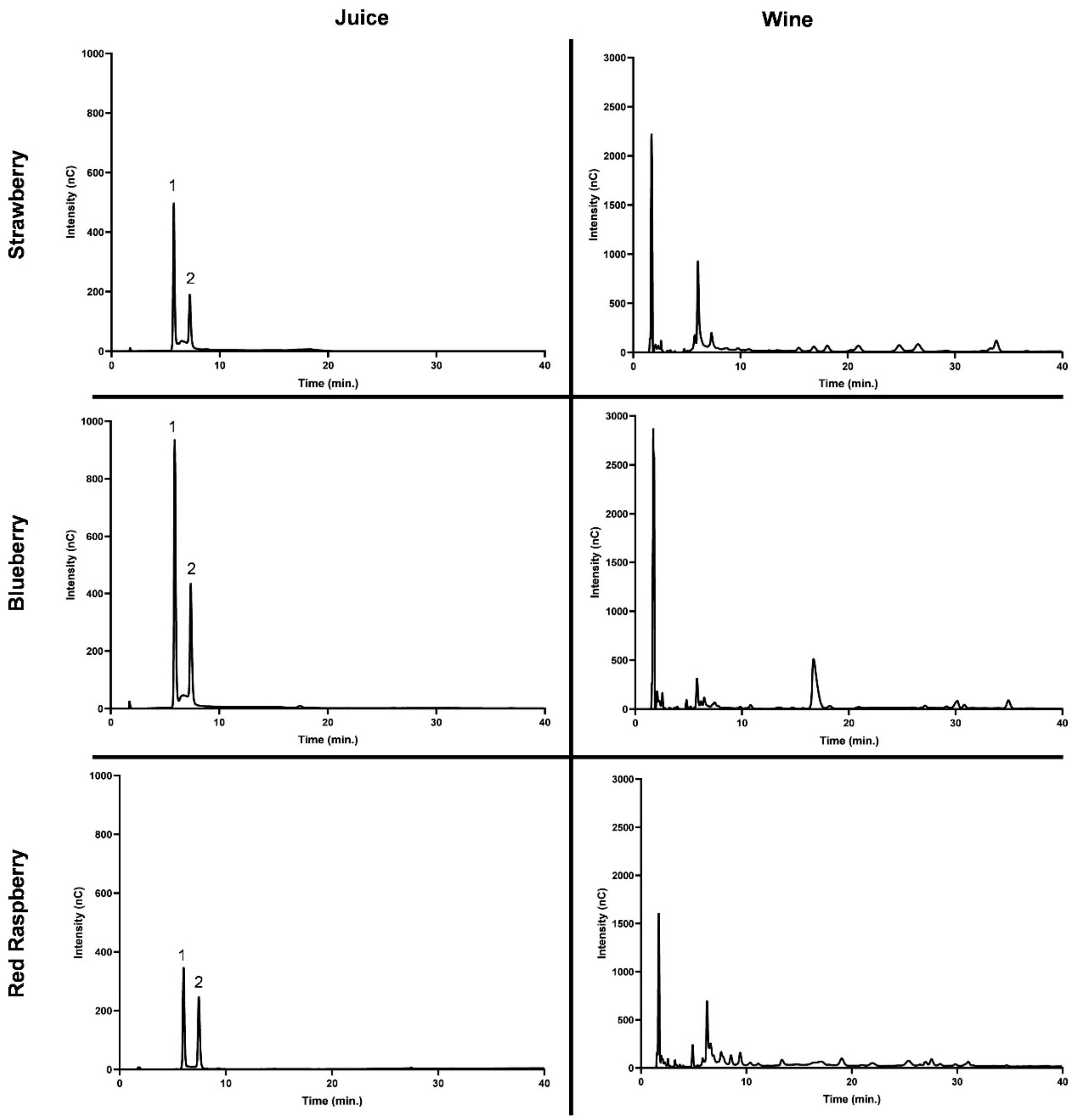

Supplementary Figure S1 HPAEC-PAD chromatograms of strawberry, blueberry and red raspberry juices (dilution 1:500) and wines (dilution 1:2). Glucose (1) and fucose (2) were identified by comparison with commercial pure standards.

Supplementary Table S1 Ethanol concentration (% v/v) of red raspberry, blueberry, and strawberry was determined for each100 mL distilled fraction until reaching 0.00 % v/v. The values are represented as mean ± standard deviation (n=2) of ethanol content.

Fraction	Red Raspberry			Strawberry			Blueberry		
	mean	sd	n	mean	sd	n	mean	sd	n
1	38.277	0.268	2	39.029	0.319	2	49.808	0.024	2
2	34.955	0.252	2	33.607	0.107	2	44.737	0.031	2
3	31.835	0.257	2	30.796	0.045	2	42.596	0.099	2
4	28.119	1.022	2	28.399	0.005	2	40.059	0.351	2
5	25.402	1.015	2	25.915	0.122	2	37.227	0.786	2
6	22.702	1.346	2	23.12	0.661	2	34.838	0.763	2
7	19.970	1.423	2	21.013	0.174	2	32.104	0.917	2
8	17.601	1.366	2	18.756	0.223	2	29.073	0.905	2
9	15.252	1.279	2	16.611	0.110	2	26.329	0.783	2
10	13.195	1.139	2	14.670	0.157	2	23.906	0.861	2
11	11.084	0.859	2	12.780	0.050	2	21.534	0.687	2
12	9.431	0.748	2	11.106	0.097	2	19.286	0.989	2
13	8.032	0.655	2	9.538	0.044	2	17.326	0.853	2
14	6.878	0.583	2	8.153	0.102	2	15.590	0.600	2
15	5.723	0.534	2	6.826	0.060	2	13.562	0.807	2
16	4.734	0.507	2	5.799	0.047	2	11.849	0.771	2
17	3.895	0.444	2	4.850	0.032	2	10.090	0.637	2
18	3.193	0.443	2	4.093	0.004	2	8.690	0.939	2
19	2.565	0.395	2	3.373	0.032	2	7.209	0.780	2
20	2.058	0.380	2	2.787	0.024	2	6.002	0.687	2
21	1.660	0.300	2	2.294	0.022	2	4.992	0.597	2
22	1.326	0.265	2	1.850	0.020	2	4.128	0.487	2
23	1.058	0.229	2	1.504	0.018	2	3.364	0.393	2
24	0.864	0.176	2	1.199	0.006	2	2.745	0.305	2
25	0.666	0.160	2	0.957	0.015	2	2.306	0.266	2
26	0.514	0.131	2	0.759	0.012	2	1.872	0.214	2
27	0.395	0.104	2	0.596	0.014	2	1.516	0.207	2
28	0.296	0.090	2	0.455	0.005	2	1.196	0.164	2
29	0.215	0.074	2	0.341	0.001	2	0.962	0.127	2
30	0.152	0.057	2	0.254	0.000	2	0.819	0.024	2
31	0.104	0.045	2	0.185	0.003	2	0.640	0.015	2
32	0.064	0.036	2	0.128	0.003	2	0.475	0.004	2
33	0.030	0.030	2	0.083	0.003	2	0.370	0.012	2
34	0.031	0.000	2	0.049	0.004	2	0.274	0.010	2
35	0.008	0.000	2	0.026	0.007	2	0.200	0.010	2
36	0.000	0.000	2	0.004	0.004	2	0.149	0.005	2
37	-	-	-	0.000	0.000	2	0.105	0.000	2
38	-	-	-	-	-	-	0.071	0.001	2
39	-	-	-	-	-	-	0.000	0.000	2

Supplementary Table S2 Concentration (mg/L) of major congeners and ethanol content (% v/v) of red raspberry, blueberry, and strawberry in each 100 mL distilled fraction. The values are represented as mean ± standard deviation (n=2), and the distillation cuts are represented as “heart” or “tail” fraction.

		**Acetaldehyde**			**Ethyl Acetate**			**Methanol**			**Propanol**			**Isobutanol**			**3-Metil_Butanol**			**Ethanol (% v/v)**		
		**mean**	**sd**	**n**	**mean**	**sd**	**n**	**mean**	**sd**	**n**	**mean**	**sd**	**n**	**mean**	**sd**	**n**	**mean**	**sd**	**n**	**mean**	**sd**	**n**
**Red Raspberry**																						
**heart**	1	917,637	0,174	2	1485,302	0,281	2	2899,719	0,587	2	459,898	0,087	2	409,500	0,068	2	717,768	0,096	2	38,277	0.268	2
	2	517,530	0,098	2	852,669	0,162	2	2325,502	0,441	2	355,682	0,067	2	280,369	0,049	2	500,779	0,079	2	34,956	0.2525	2
**tail**	3	182,651	0,035	2	537,689	0,102	2	2472,506	0,469	2	342,763	0,065	2	217,170	0,039	2	394,577	0,066	2	31.835	0.257	2
	4	194,213	0,037	2	550,017	0,104	2	2494,931	0,473	2	367,498	0,070	2	203,462	0,036	2	328,900	0,056	2	28.119	1.022	2
	5	119,747	0,023	2	247,474	0,047	2	2517,796	0,477	2	216,190	0,041	2	112,312	0,021	2	192,394	0,035	2	25.402	1.015	2
	6	57,235	0,011	2	435,019	0,082	2	1654,932	0,314	2	173,184	0,033	2	71,943	0,013	2	121,930	0,022	2	22.7025	1.3465	2
	7	54,013	0,010	2	365,526	0,069	2	1918,383	0,364	2	156,153	0,030	2	53,226	0,010	2	82,017	0,015	2	19.97	1.423	2
	8	27,036	0,005	2	594,666	0,113	2	1779,456	0,337	2	144,395	0,027	2	39,478	0,007	2	58,122	0,011	2	17.6015	1.3665	2
	9	34,475	0,007	2	376,101	0,071	2	1918,832	0,364	2	115,555	0,022	2	24,363	0,005	2	34,422	0,006	2	15.252	1.279	2
	10	29,117	0,006	2	587,646	0,111	2	1103,974	0,209	2	143,149	0,027	2	21,968	0,004	2	27,837	0,005	2	13.1955	1.1395	2
	11	31,835	0,006	2	471,307	0,089	2	1995,546	0,378	2	90,833	0,017	2	10,634	0,002	2	13,510	0,003	2	11.0845	0.8595	2
	12	22,151	0,004	2	179,034	0,034	2	1410,207	0,267	2	45,591	0,009	2	5,133	0,001	2	5,486	0,001	2	9.4315	0.7485	2
	13	14,481	0,003	2	297,438	0,056	2	1127,855	0,214	2	34,217	0,006	2	2,578	0,000	2	0,296	0,000	2	8.032	0.655	2
	14	33,305	0,006	2	471,454	0,089	2	866,266	0,164	2	25,949	0,005	2	-	-	-	2,174	0,000	2	6.8785	0.5835	2
	15	18,949	0,004	2	629,030	0,119	2	555,483	0,105	2	24,090	0,005	2	-	-	-	-	-	-	5.723	0.534	2
	16	13,669	0,003	2	483,503	0,092	2	463,004	0,088	2	15,331	0,003	2	-	-	-	-	-	-	4.734	0.507	2
	17	13,374	0,003	2	489,456	0,093	2	408,181	0,077	2	16,267	0,003	2	-	-	-	-	-	-	3.895	0.444	2
	18	12,788	0,002	2	104,822	0,072	2	676,002	0,066	2	5,514	0,001	2	-	-	-	-	-	-	3.1935	0.4435	2
	19	11,111	0,004	2	380,654	0,073	2	348,441	0,083	2	6,625	0,001	2	-	-	-	-	-	-	2.565	0.395	2
	20	21,885	0,003	2	383,167	0,023	2	437,466	0,089	2	6,959	0,000	2	-	-	-	-	-	-	2.058	0.38	2
	21	15,879	0,003	2	120,480	0,053	2	470,716	0,051	2	-	-	-	-	-	-	-	-	-	1.6605	0.3005	2
	22	17,970	0,003	2	279,042	0,035	2	269,244	0,038	2	-	-	-	-	-	-	-	-	-	1.3265	0.2645	2
	23	14,204	0,001	2	185,530	0,023	2	202,898	0,037	2	-	-	-	-	-	-	-	-	-	1.058	0.229	2

	24	7,113	0,004	2	119,820	0,029	2	197,651	0,050	2	-	-	-	-	-	-	-	-	-	0.864	0.176	2
	25	18,480	0,004	2	155,350	0,019	2	262,533	0,005	2	-	-	-	-	-	-	-	-	-	0.6665	0.1605	2
	26	18,884	0,002	2	139,658	0,025	2	118,301	0,026	2	-	-	-	-	-	-	-	-	-	0.5145	0.1315	2
	27	11,884	0,002	2	132,902	0,022	2	139,150	0,017	2	-	-	-	-	-	-	-	-	-	0.3955	0.1045	2
	28	8,429	0,000	2	113,894	0,021	2	90,486	0,023	2	-	-	-	-	-	-	-	-	-	0.296	0.09	2
	29	1,578	0,004	2	110,257	0,018	2	121,091	0,018	2	-	-	-	-	-	-	-	-	-	0.215	0.074	2
	30	21,325	0,000	2	95,155	0,026	2	93,893	0,022	2	-	-	-	-	-	-	-	-	-	0.1525	0.0575	2
	31	-	-	-	100,746	0,011	2	127,797	0,019	2	-	-	-	-	-	-	-	-	-	0.104	0.045	2
	32	-	-	-	56,616	0,011	2	101,755	0,020	2	-	-	-	-	-	-	-	-	-	0.0645	0.0365	2
	33	-	-	-	56,109	0,015	2	106,321	0,024	2	-	-	-	-	-	-	-	-	-	0.0305	0.0305	2
	34	-	-	-	66,775	0,013	2	66,960	0,013	2	-	-	-	-	-	-	-	-	-	0.031	0,000	2
	35	-	-	-	57,922	0,019	2	60,933	0,019	2	-	-	-	-	-	-	-	-	-	0.008	0,000	2
**Blueberry**																						
	1	321,417	0,061	2	193,044	0,037	2	487,997	0,092	2	309,074	0,059	2	451,206	0,073	2	1002,358	0,061	2	49,809	0,025	2
	2	312,834	0,059	2	174,697	0,033	2	742,349	0,141	2	311,631	0,059	2	393,710	0,066	2	1002,460	0,047	2	44,737	0,031	2
	3	224,261	0,042	2	271,847	0,052	2	644,106	0,122	2	288,421	0,055	2	346,344	0,059	2	942,726	0,078	2	42,596	0,099	2
**heart**	4	87,301	0,017	2	123,524	0,023	2	723,842	0,137	2	255,997	0,049	2	266,438	0,047	2	860,873	0,093	2	40,059	0,351	2
	5	78,129	0,015	2	133,142	0,025	2	674,336	0,128	2	211,512	0,040	2	193,379	0,035	2	626,129	0,091	2	37,227	0,786	2
	6	42,457	0,008	2	107,803	0,020	2	776,835	0,147	2	175,424	0,033	2	140,949	0,026	2	464,947	0,075	2	34,838	0,763	2
	7	27,631	0,005	2	182,969	0,035	2	449,074	0,085	2	167,134	0,032	2	107,641	0,020	2	360,057	0,061	2	32,105	0,918	2
	8	17,311	0,003	2	121,411	0,023	2	634,223	0,120	2	160,591	0,030	2	95,201	0,018	2	304,143	0,053	2	29,073	0,905	2
	9	6,660	0,001	2	99,736	0,019	2	589,068	0,112	2	119,863	0,023	2	52,584	0,010	2	168,837	0,031	2	26,330	0,784	2
	10	5,612	0,001	2	98,775	0,019	2	520,333	0,099	2	95,127	0,018	2	36,878	0,007	2	110,890	0,020	2	23,906	0,861	2
	11	5,924	0,001	2	97,601	0,018	2	476,324	0,090	2	80,347	0,015	2	27,707	0,005	2	86,361	0,016	2	21,534	0,687	2
**tail**	12	-	-	-	90,887	0,017	2	450,049	0,085	2	65,440	0,012	2	17,551	0,003	2	51,614	0,010	2	19,286	0,989	2
	13	-	-	-	162,098	0,031	2	320,878	0,061	2	60,549	0,011	2	12,546	0,002	2	36,826	0,007	2	17,327	0,854	2
	14	-	-	-	99,153	0,019	2	402,767	0,076	2	39,421	0,007	2	4,612	0,001	2	13,524	0,003	2	15,591	0,601	2
	15	-	-	-	91,997	0,017	2	369,693	0,070	2	33,504	0,006	2	3,198	0,001	2	10,894	0,002	2	13,562	0,807	2
	16	-	-	-	140,234	0,027	2	341,898	0,065	2	28,916	0,005	2	0,000	0,000	2	6,723	0,001	2	11,850	0,772	2

	17	-	-	-	128,318	0,024	2	261,925	0,050	2	20,568	0,004	2	-	-	-	3,340	0,001	2	10,091	0,638	2
	18	-	-	-	99,783	0,019	2	248,101	0,047	2	14,571	0,003	2	-	-	-	-	-	-	8,690	0,939	2
	19	-	-	-	113,461	0,022	2	161,774	0,031	2	10,007	0,002	2	-	-	-	-	-	-	7,210	0,781	2
	20	-	-	-	108,387	0,021	2	134,979	0,026	2	8,562	0,002	2	-	-	-	-	-	-	6,003	0,688	2
	21	-	-	-	71,763	0,014	2	175,415	0,033	2	-	-	-	-	-	-	-	-	-	4,993	0,598	2
	22	-	-	-	68,166	0,013	2	132,418	0,025	2	-	-	-	-	-	-	-	-	-	4,129	0,488	2
	23	-	-	-	73,001	0,014	2	126,905	0,024	2	-	-	-	-	-	-	-	-	-	3,364	0,393	2
	24	-	-	-	70,511	0,013	2	105,350	0,020	2	-	-	-	-	-	-	-	-	-	2,746	0,306	2
	25	-	-	-	76,022	0,014	2	69,125	0,013	2	-	-	-	-	-	-	-	-	-	2,306	0,266	2
	26	-	-	-	60,081	0,011	2	71,436	0,014	2	-	-	-	-	-	-	-	-	-	1,873	0,215	2
	27	-	-	-	40,907	0,008	2	81,254	0,015	2	-	-	-	-	-	-	-	-	-	1,516	0,207	2
	28	-	-	-	0,000	0,000	2	111,614	0,021	2	-	-	-	-	-	-	-	-	-	1,197	0,165	2
	29	-	-	-	29,921	0,006	2	51,670	0,010	2	-	-	-	-	-	-	-	-	-	0,963	0,128	2
	30	-	-	-	0,000	0,000	2	92,960	0,018	2	-	-	-	-	-	-	-	-	-	0,820	0,025	2
	31	-	-	-	23,347	0,004	2	37,244	0,007	2	-	-	-	-	-	-	-	-	-	0,640	0,015	2
	32	-	-	-	19,359	0,004	2	34,186	0,006	2	-	-	-	-	-	-	-	-	-	0,476	0,005	2
	33	-	-	-	37,429	0,007	2	106,471	0,020	2	-	-	-	-	-	-	-	-	-	0,371	0,013	2
	34	-	-	-	12,836	0,002	2	35,063	0,007	2	-	-	-	-	-	-	-	-	-	0,274	0,010	2
	35	-	-	-	19,853	0,004	2	30,916	0,006	2	-	-	-	-	-	-	-	-	-	0,201	0,011	2
	36	-	-	-	17,453	0,003	2	20,510	0,004	2	-	-	-	-	-	-	-	-	-	0,149	0,005	2
	37	-	-	-	11,737	0,002	2	14,906	0,003	2	-	-	-	-	-	-	-	-	-	0,106	0,001	2
	38	-	-	-	6,966	0,002	2	15,787	0,004	2	-	-	-	-	-	-	-	-	-	0,072	0,002	2
**Strawberry**																						
**heart**	1	428,301	0,081	2	707,315	0,134	2	3432,834	0,651	2	473,333	0,090	2	421,590	0,069	2	1022,061	0,004	2	39,030	0,320	2
**tail**	2	333,810	0,063	2	703,262	0,133	2	4261,978	0,808	2	384,605	0,073	2	264,903	0,046	2	1005,490	0,044	2	33,608	0,108	2
	3	140,836	0,027	2	694,163	0,132	2	3098,252	0,587	2	378,899	0,072	2	119,763	0,701	2	1005,826	0,043	2	30,796	0,045	2
	4	114,558	0,022	2	691,942	0,131	2	4211,224	0,798	2	362,746	0,069	2	205,002	0,037	2	691,974	0,095	2	28,400	0,006	2

5	70,551	0,013	2	414,912	0,079	2	2401,154	0,455	2	212,460	0,040	2	114,955	0,021	2	389,495	0,065	2	25,916	0,123	2
6	32,425	0,006	2	512,817	0,097	2	1715,427	0,325	2	168,664	0,032	2	76,878	0,014	2	261,984	0,046	2	23,120	0,661	2
7	20,423	0,004	2	717,600	0,136	2	1073,772	0,203	2	145,145	0,028	2	53,287	0,010	2	175,939	0,032	2	21,014	0,175	2
8	38,042	0,007	2	554,202	0,105	2	1364,079	0,258	2	117,747	0,022	2	35,995	0,007	2	114,329	0,021	2	18,756	0,223	2
9	-	-	-	317,677	0,060	2	1313,202	0,249	2	113,749	0,022	2	27,445	0,005	2	81,853	0,015	2	16,612	0,111	2
10	-	-	-	399,045	0,076	2	1690,361	0,320	2	89,730	0,017	2	16,762	0,003	2	47,431	0,009	2	14,671	0,158	2
11	-	-	-	296,243	0,056	2	1593,245	0,302	2	63,727	0,012	2	12,462	0,002	2	36,284	0,007	2	12,781	0,051	2
12	-	-	-	256,861	0,049	2	1144,092	0,217	2	70,012	0,013	2	8,646	0,002	2	19,703	0,004	2	11,107	0,098	2
13	-	-	-	168,897	0,032	2	292,233	0,055	2	60,861	0,012	2	14,459	0,003	2	37,365	0,007	2	9,538	0,044	2
14	-	-	-	402,028	0,076	2	1212,586	0,230	2	36,204	0,007	2	6,784	0,001	2	6,348	0,001	2	8,153	0,102	2
15	-	-	-	424,873	0,081	2	1083,929	0,205	2	59,467	0,011	2	-	-	-	-	-	-	6,826	0,060	2
16	-	-	-	477,873	0,091	2	776,624	0,147	2	11,810	0,002	2	-	-	-	-	-	-	5,799	0,047	2
17	-	-	-	297,438	0,048	2	587,725	0,148	2	-	-	-	-	-	-	-	-	-	4,851	0,033	2
18	-	-	-	283,321	0,041	2	505,961	0,183	2	-	-	-	-	-	-	-	-	-	4,093	0,004	2
19	-	-	-	231,993	0,056	2	337,977	0,111	2	-	-	-	-	-	-	-	-	-	3,373	0,032	2
20	-	-	-	298,871	0,054	2	190,884	0,096	2	-	-	-	-	-	-	-	-	-	2,787	0,024	2
21	-	-	-	77,625	0,044	2	98,562	0,064	2	-	-	-	-	-	-	-	-	-	2,295	0,023	2
22	-	-	-	161,986	0,057	2	172,225	0,036	2	-	-	-	-	-	-	-	-	-	1,850	0,020	2
23	-	-	-	137,715	0,015	2	180,925	0,019	2	-	-	-	-	-	-	-	-	-	1,505	0,019	2
24	-	-	-	153,643	0,031	2	130,786	0,033	2	-	-	-	-	-	-	-	-	-	1,199	0,006	2
25	-	-	-	134,893	0,026	2	115,193	0,034	2	-	-	-	-	-	-	-	-	-	0,958	0,016	2
26	-	-	-	100,199	0,029	2	131,562	0,025	2	-	-	-	-	-	-	-	-	-	0,760	0,013	2
27	-	-	-	91,784	0,026	2	71,569	0,022	2	-	-	-	-	-	-	-	-	-	0,596	0,014	2
28	-	-	-	76,689	0,019	2	62,726	0,025	2	-	-	-	-	-	-	-	-	-	0,455	0,005	2
29	-	-	-	45,968	0,017	2	80,941	0,014	2	-	-	-	-	-	-	-	-	-	0,341	0,001	2
30	-	-	-	57,713	0,015	2	45,268	0,012	2	-	-	-	-	-	-	-	-	-	0,255	0,001	2
31	-	-	-	56,987	0,009	2	78,744	0,015	2	-	-	-	-	-	-	-	-	-	0,185	0,004	2
32	-	-	-	57,603	0,011	2	45,183	0,009	2	-	-	-	-	-	-	-	-	-	0,129	0,004	2
33	-	-	-	56,879	0,011	2	78,594	0,015	2	-	-	-	-	-	-	-	-	-	0,084	0,004	2

34	-	-	-	19,563	0,193	2	15,268	0,203	2	-	-	-	-	-	-	-	-	-	0,050	0,005	2
35	-	-	-	10,068	0,193	2	11,068	0,203	2	-	-	-	-	-	-	-	-	-	0,026	0,007	2
36	-	-	-	10,043	0,202	2	10,329	0,192	2	-	-	-	-	-	-	-	-	-	0,005	0,005	2

- not detected
